# Comparative clinical and cost analysis between surgical and non-surgical intervention for knee osteoarthritis

**DOI:** 10.1007/s00264-019-04405-y

**Published:** 2019-09-13

**Authors:** Luxme Mahendira, Caroline Jones, Angelo Papachristos, James Waddell, Laurence Rubin

**Affiliations:** 1 Division of Rheumatology, Unity Health-Saint Michael’s Hospital, 30 Bond Street, Bond Wing 3-061, Toronto, Ontario M5B 1W8 Canada; 2 Mobility Program, Unity Health-Saint Michael’s Hospital, 30 Bond Street, Toronto, Ontario M5B 1W8 Canada; 3 Faculty of Medicine, University of Toronto, Toronto, M5S 1A8 Canada; 4 Division of Orthopedics, Unity Health-Saint Michael’s Hospital, 55 Queen St E. Suite 207, Toronto, Ontario M5C 1R6 Canada

**Keywords:** Osteoarthritis, Arthritis, Total knee arthroplasty, Multidiscplinary medical management, Health economics, Models of care, Comprehensive non-surgical management, WOMAC, Advanced clinician practitioner, Advanced practice physcial therapist

## Abstract

**Objective:**

To evaluate the management and costs of osteoarthritis of the knee (OAK), a progressive joint disease due to bone and cartilage degeneration, with significant personal and societal impact.

**Methods:**

We prospectively analyzed the clinical outcomes and quantifiable cumulative direct costs of patients with OAK referred to our multidisciplinary OA program over a two year time period. One hundred thirty-one subjects were assessed. All demonstrated radiographic criteria for moderate to severe OAK. Western Ontario McMaster Osteoarthritis Index (WOMAC), Minimal Clinically Important Improvement (MCII), and change in BMI were recorded and analyzed. Total medical and surgical direct costs for all subjects during the two year time period were determined.

**Results:**

Five patients underwent total joint replacement during the two years of study. Among the group as a whole, a significant overall improvement in WOMAC scores was noted at the two year time point follow-up. After dividing the group into tertiles by baseline WOMAC scores, 46% achieved MCII. Significant weight loss was noted for individuals with baseline BMI of > 30. As all patients were considered “de facto” surgical candidates at referral, an average net savings of $9551.10 of direct costs per patient, or a potential total of $1,203,438.60 for the entire group, could be inferred as a result of medical as opposed to surgical management.

**Conclusion:**

These findings support the benefits of multidisciplinary medical management for patients with significant OAK. This approach is clinically beneficial and may provide significant cost savings. Such models of care can substantially improve the long-term outcome of this highly prevalent condition and reduce societal and financial burdens.

**Electronic supplementary material:**

The online version of this article (10.1007/s00264-019-04405-y) contains supplementary material, which is available to authorized users.

## Introduction

Osteoarthritis (OA) is a progressive joint disease due to bone and cartilage degeneration, with a significant personal and societal impact. Costs associated with OA management involve not only direct treatment strategies (clinic assessments, medications, orthotics, surgery), but also significant indirect costs (loss of productivity of patient and caregivers through both physical and mental burden) [1–6]. Approximately one in four Canadians suffer from osteoarthritis, with a growing economic burden estimated, through direct and indirect costs associated with this disease, in 2010 at $27.5 Billion and increasing by 2040 to almost $1.5 trillion [1]. Approximately 30% of the Canadian labor force is expected in the coming decade to experience pain related to osteoarthritis, which often leads to disability [1, 7, 8]. With the increasing incidence rates of OA, effective short- and long-term management strategies are required to cope with this disease.

Osteoarthritis of the knee (OAK) is the most common type of OA with affecting up to 19% of adults (age 45 and older) according to recent reviews [9]. The severity of OAK symptoms and required intervention can vary. Contributing modifiable factors to OAK severity include increasing rates of obesity, insufficient patient education, limited access to OA health care providers, and poor pain management strategies [4–10].

Both surgical and non-surgical treatment strategies are implemented for OAK. Total joint replacement (TJR) is a surgical option for severe OAK and has been shown to provide long-term benefit [11]. However, not all individuals with OAK can or even wish to proceed with surgery. Firstly, patients may not yet have acquired significant disease burden to warrant the potential complications associated with surgery. Additionally, the existence of significant medical co-morbidities (e.g., severe obesity, COPD) may preclude eligibility for TJR. Additionally, access to surgical intervention may be limited or delayed in many jurisdictions due to budgetary restrictions and limited resources (operating time and surgeon availability). Finally, there can be significant pain and potential lack of efficacy following TJR, which ultimately may impact an individual’s decision to consider this option [11].

Because surgical intervention is not always the preferred or available treatment strategy for individuals with OAK, there is an increasing emphasis on the optimization of modifiable OAK risk factors, as they have been shown to significantly reduce pain and disability [12, 13]. Other effective treatment strategies for OAK include biomechanical interventions, intra-articular corticosteroids and other injectable preparations, anti-inflammatory and analgesic medications, exercise (water and land based), patient education, and strength training [14]. Multidisciplinary models of care for non-surgical OAK treatment include the contributions of physiotherapists, occupational therapists, and rheumatologists, with the emphasis placed on patient education regarding weight loss, exercise, and pain management have been implemented in various countries [15]. We sought to determine if such interventions would have benefit on our population of OAK patients who were referred initially to our orthopedic service for consideration of joint replacement. These models of care have been shown to have a positive effect on reduction on overall wait-times for initial assessment, but they are still underutilized.

## Methods

Approval for this study was obtained from our institutional REB.

### Study population

St. Michael’s Hospital (SMH) is an academic medical center that offers both surgical and non-surgical intervention for OAK. Individuals considered for TJR, as well as those deemed not yet surgical candidates, are evaluated and followed in our multidisciplinary osteoarthritis practice. Our patient-centered model of care includes rheumatologists, physiotherapists, and nurses, with access to occupational therapists and weight management options, including referral where appropriate for assessment for bariatric surgery. We employ a comprehensive non-surgical treatment plan to help improve function and decrease pain, including intra-articular corticosteroid and hyaluronic acid injections, non-steroidal anti-inflammatory agents, and standardized exercise programs, with a focus on muscle strengthening and coordination, both individualized and group physiotherapy. Standardized measures of objective and subjective severity of OAK-related pain and disability are documented at each patient visit.

All patients deemed to be arthroplasty candidates who were initially referred to one of our orthopaedic arthroplasty surgeons (JW) and who demonstrated baseline radiographs of at least moderate to severe osteoarthritis of the knee (as defined by expert musculoskeletal radiologists at our centre) were followed for a total of two years in the OA program or until referred for joint replacement surgery.

### Study measures

Age and gender, BMI, and Western Ontario and McMaster Universities Osteoarthritis Index (WOMAC) [16] scores at baseline and at each subsequent visit over the two years were recorded. The average change from baseline to two years in BMI and WOMAC scores for the group was calculated.

### Minimally clinical important improvement (MCII)

The study group was also divided into tertiles based on their baseline WOMAC score: low [< 35], medium [36–51], and high [> 52–96]. We then evaluated each group’s response over the two year time frame by calculating the number of individuals who achieved a minimally clinical important improvement (MCII) for each tertile. However, rather than analyzing the WOMAC subscales as previously reported [17], we applied these same thresholds to the composite WOMAC score.

### Medical and surgical costs

All non-surgical and surgical costs (in Canadian dollars) were determined and totaled for each patient regardless of outcome. Non-surgical costs included consultant assessment fees, analgesics, corticosteroids and hyaluronic acid preparations for intra-articular injection, and disposable equipment (syringes, needles, alcohol wipes, and band aids). Surgical costs included consultant assessment fees for each visit as well as preoperative assessments, hospital admission costs, surgical instrumentation (including prosthetic joint materials), and post-operative rehabilitation. These costs are similar to costs at other Ontario hospitals performing high rates of TJR.

We also calculated a hypothetical surgical cost for the entire group given that all subjects, at the time of referral, were deemed to be surgical candidates for TJR.

### Statistical analyses

Univariate analyses of the change in WOMAC scores and BMI were determined. For BMI, initial vs. final visit were analyzed. For the BMI analyses, we further analyzed the change for that subset of individuals whose baseline BMI was > 30.

## Results

One hundred thirty-one subjects met radiographic criteria for moderate to severe OAK (46 moderate, 45 mod/severe, 40 severe). Among the 131 individuals, 97 were women (74%). The average age was 66.2 years (range 38 to 93 years). The average BMI was 35.22, with a decrease in average final BMI to 32.42. Of the 131 subjects, 73 had an initial BMI of equal to or greater than 30.

The change in BMI from baseline to 2 years was not statistically significant (mean change = − 0.84, *p* = 0.1, 95% CI = − 1.31 to − 0.37). However, for those subjects with a baseline BMI of greater than 30, the change in BMI at the end of the 2-year period was significant (mean change = − 0.95, *p* = 0.05, 95% CI − 1.24 to − 0.44) (Table 1).

**Table 1:** Patient characteristics (*n* = 131), WOMAC scores, BMI, and change over the 2 years

Category	Results
Female (%)	97(74%)
Average age years (range)	66.2(38–93)
Average BMI at entry Average BMI at 2 years Change in BMI at 2 years Number of pts. with BMI > 30 at entry Change in BMI at 2 years	34.2 32.4 − 0.84 (*p* = 0.1, CI − 1.31 to − 0.37) 72 − 0.95 (*p* = 0.05, CI − 1.24 to − 0.44)
Baseline WOMAC (range)	45.2(7–88)
Average WOMAC at 2 years (range)	37 (4–69)
Number of TJR	5

**Fig. 1:**
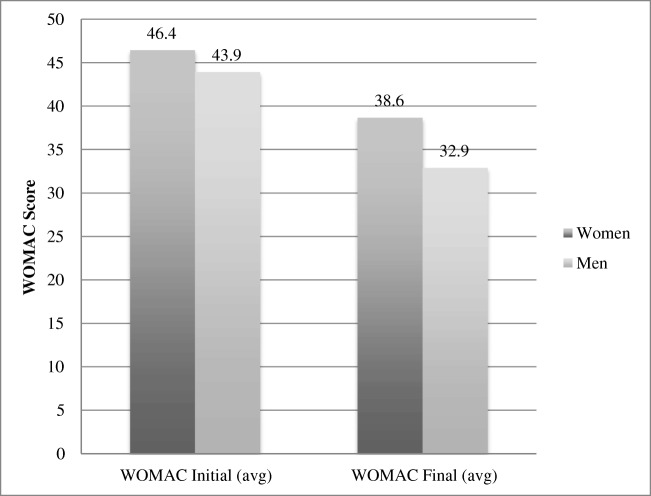
Average change in WOMAC score form baseline to end of two years

The average change in WOMAC scores from baseline to the end of the two years is shown in Fig. 1. For the group as a whole, a significant improvement in WOMAC scores was noted at the end of the two year follow-up. The average baseline WOMAC score was 45.82, (range 7 to 88). 63.6% of individuals (83/131) had a WOMAC score > 39. At two years, the mean final WOMAC score was 37 (range 4 to 69) (*p* = 0.05, 95% CI 11.4–16.1, Table 1).

**Fig. 2:**
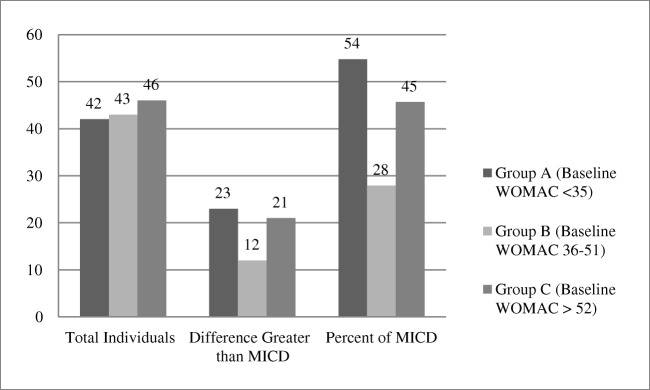
Tertiles by baseline WOMAC scores

After dividing the group into tertiles by baseline WOMAC scores, 46% achieved MCII. The number of subjects in the highest and lowest tertiles achieved this threshold at a much greater frequency than the middle group (Fig. 2).

Over the two year time frame, 5/131 patients proceeded to TJR. Three were women (age 53, 60 and 81) and two men (age 55 and 77). All individuals had baseline radiographs demonstrating severe OA. Of note, the baseline average WOMAC score did not differ from the group as a whole (44, range 24–70). The final recorded WOMAC score pre-operatively did not change significantly from baseline in 4/5 patients (average 43, range 19–66).

### Medical and surgical costs

The total cost of assessments, investigations, and procedures including all surgical and non-surgical interventions are detailed in Appendices 1 and 2 (recorded in Canadian Dollars).

**Fig. 3:**
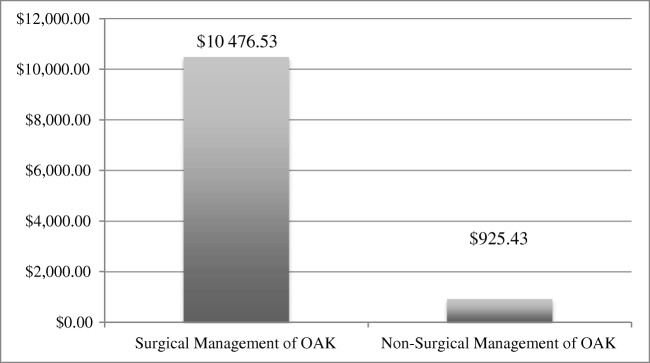
The average surgical costs

The average surgical costs (pre-operative, operative, and post-operative expenses) of the five patients who underwent arthroplasty were $10,476.53, with the majority of surgery-related cost associated with the operation, hospitalization and post op care. The average cost of medical management over the two year time frame (including the five who ultimately proceeded to surgery) was $925.43 (Fig. 3).

Given that all patients were considered as surgical candidates at the time of initial referral to our program, we potentially achieved an average net savings of $9551.10 of direct costs per patient, or a total of $1,203,438.60 for the entire cohort over the two years of follow-up.

## Discussion

The burden of OAK on both the individual and society is high, encompassing physical, mental, and economic strain. A proactive approach to managing OAK is imperative to improve quality of life, as well as to mitigate health care costs.

There has yet to be a detailed Canadian analysis of the financial impact of surgical versus non-surgical interventions in OAK. To quantify the medical and economic benefit of the OAK program at our institution, we chose to analyze the clinical outcomes and costs for surgical and non-surgical intervention for OAK in a group of patients who, under current community standards, would be deemed surgical candidates. All these individuals were initially referred to an orthopedic arthroplasty program for assessment, had significant radiographic changes, and had failed standard interventions offered in the typical community setting.

We have demonstrated clinical benefit and highly favourable cost savings in the treatment of OAK over a two year period through the utilization of non-surgical interventions in a structured multidisciplinary OA program. The medical management group demonstrated a significant improvement with their pain and function, as illustrated by WOMAC score improvement and mitigation of the need for TJR, at least during this two year period of active intervention, and possibly beyond.

Our study represents one of the longest recorded prospective and real world analyses of OAK knee management that we are aware of; previous studies that have assessed shorter intervals, or analyzed only surgical outcomes, do not report on subsequent surgical outcomes in medically managed cohorts, nor provide any direct and detailed cost comparisons. We have also found that our professional colleagues, both medical and allied health personnel, as well as our patients, are unaware of the costs of these assessments, tests and interventions. Such data can play an important role in educating the profession and the public on the cost of OAK management, and to an informed discussion regarding health care resource allocation.

Our cost analysis was performed from a Canadian perspective. Recent U.S. reports estimated total knee replacement costs on average at $50,000 USD [18], which on a cost adjusted basis translates to a 7.5 times greater cost/TJR in the US versus Ontario. While our health care systems do differ in some significant ways, this difference in cost is still much greater than would be expected and warrants further evaluation. There may be jurisdictions in the US more in line with our costs, but the variability is still very substantial and to our knowledge does not reflect any difference in efficacy or overall outcomes [19].

In a study comparing the outcome of modern and traditional knee implants using the Short Form 6 dimensional (SF-6D) score and quality adjusted life year (QALY) methodology, the modern implant was found to cost $497.66 (£298 in November 2013) less per QALY at one year [20]. As technology continues to progress, we can expect TKA to become less expensive and more effective, making surgery a more cost-effective and clinically effective approach than it is today.

Though all subjects were initially referred to our orthopaedic service for consideration of arthroplasty, the number of patients in this cohort who ultimately proceeded to joint replacement was very small (5/131 or 3.8%). No general conclusions can be made to better identify those patients at baseline who would inevitably proceed to arthroplasty. The average WOMAC score at baseline did not differ between these individuals and the group as a whole. In contrast to the improvements noted in most of the subjects, four of the five individuals who proceeded to joint replacement demonstrated no significant reduction in their final preoperative recorded WOMAC. The average BMI at enrollment was lower in these five individuals as compared to the entire cohort (29 versus 34), but the average and individual values did not change at all from baseline to the last measure before surgery.

While some patients may have been unwilling to undergo joint replacement surgery despite a recommendation to do so, due to age, perception of risk, and co-morbidity, the basis for this decision, in our experience, can change from visit to visit. Others may not be “true” surgical candidates, despite an initial referral to orthopedics and radiographic features. Symptoms and limitations may fluctuate, regardless of intervention. However, none of these factors likely impacts the overall outcome we observed during this extended follow up period. Even if some additional subjects were eventually to proceed to joint replacement over subsequent years, we would still have realized a substantial costs savings and overall benefit for the majority. Longer-term follow-up may help clarify this outcome, but these results should not be interpreted as merely deferring an inevitable outcome (TJR). Instead, we believe that patients, who improved or even stabilized throughout this process, will be less likely in the future to consider TJR. They will also be in much better condition to make a truly informed decision regarding surgery.

Though our study was not a controlled trial of medical versus surgical management, recent publications have elucidated the overall benefit and impact of total knee replacements in comparison to medical management [21, 22]. In a long-term study on quality of life and costs in a U.S. cohort of patients followed up to eight years, quality of life improvements and cost benefits associated with surgical intervention can only be appreciated in the highest risk patients and support the necessity for early medical management [23]. Patients with preoperative complete joint space collapse (0 to 1 mm mJSW) achieve a significantly better WOMAC result from TKA than those with a mJSW equal to or greater than 2 mm [24].

We specifically examined the impact of weight change over the two year period and found no significant decrease in the average BMI of the cohort as a whole. However, for those patients with a BMI greater than 30, the current threshold of obesity [25], a significant reduction in BMI was noted at two years, which is consistent with reported outcomes [26]. Moreover, individual patients might have achieved some, albeit temporary, weight reduction at various points during the two year period which we could not capture, which may have contributed to their overall improved outcome.

This latter finding adds additional support to the many reports that in obese individuals, repetitive counseling can lead to weight loss and may mitigate OAK symptoms [27]. However, truly sustained weight reduction requires a comprehensive and supervised approach. To complement self as well as supervised regimes, we have utilized bariatric surgery referral for those patients who qualify for this procedure. It can be associated with significant weight reduction and would be of benefit regardless of whether the patient continued on a medical regimen only or ultimately required TJR [28].

There are several limitations of our study. We did not incorporate indirect costs, such as post op rehabilitation, given that this is age, overall function, and pre-op living arrangement dependent, and as such, is highly variable in our jurisdiction. A recent study reported 30-day and 90-day unplanned re-admission rates of 6.5% and 8.0%, respectively, following TKA [29]. Re-admission costs can be significant and were not included in this study. We also did not calculate costs incurred as a result of days off work and lost productivity for both the patient and any caregivers. This is an important element to consider with respect to global cost analysis in either the medical or surgical group. However, we propose that lower WOMAC scores are likely associated with acceptable occupational function. Furthermore, we anticipate that early and effective treatment for OAK will reduce global economic burden.

Our study did not consider social health determinants (SHD) in evaluating the management of post-operation care. In a study by Núñez-Cortés et al. [30], SHD were identified as one of multiple factors contributing to a patients comfort and health after undergoing TKA. Specifically, patients with lower education level showed a three-time higher likelihood of developing chronic post-surgical pain (CPSP) after TKA [30]. SHD could have impacted WOMAC scores for both surgical and non-surgical patients.

Patient education of OA is another factor that may influence the progression and recovery of the disease and was not considered in this study. In a short-term study evaluating a “joint school” for patients undergoing TKA, a significant increase in knowledge score for patients who completed the session was noted [31]. Increasing patients’ knowledge score may lead to reduced costs associated with knee surgery [31].

We also acknowledge that the use of standardized radiographic OA severity tools (such as the Kellgren and Lawrence grading system) might strengthen future studies. However, a decision to proceed to TJR should be based on more than radiographic findings alone. Continued collaboration with our orthopedic and imaging colleagues can enable us to recognize the subgroups of patients who may not respond as well to medical intervention and require surgical intervention sooner. However, it has been recognized that a discordance exists between radiographic status and clinical symptoms and outcome; therefore, we emphasize the need to incorporate relevant functional measures as better determinants of patient management [32, 33].

Most importantly, our study highlights the benefits of a “formal” model of care for OAK management. Our results support a multidisciplinary approach to the medical management of OAK which can improve the associated symptoms quality of life and function, while also delaying or negating the need for surgery, and all the while providing substantial systemic cost savings. Access to various arthritis specialists (rheumatologist, orthopaedic surgeons, physiotherapists, occupational therapists, nurses) in a continuous and comprehensive manner for both evaluation and management of OAK is an effective algorithm that promotes treatment success and the efficient use of health care dollars. At our institution, we are very fortunate that our rheumatologists and orthopaedic surgeons provide outpatient care in close proximity, a critical advantage to facilitating rapid referral, exchange of ideas and plans for individual patients. This model should be considered in any academic and large community musculoskeletal care programs wherever feasible. For other health care centers where access to surgical intervention may not be readily available, a stand-alone medical model of care is feasible. This in turn can lead to public policy change and resource allocation [34].

A recent study of over 2200 patients with moderate to severe OAK found that less than 1/3 had received comprehensive non-surgical management, with over 1/4 prescribed opioid medication, a pathway that may lead to significant morbidity [35]. These latter prescriptions were not correlated with pain severity, but with lower function and co-morbidity. These findings are further evidence and support for patient centered, comprehensive medical programs for treatment of OAK. Those who truly fail such efforts would then be the optimal candidates for TJR.

## Electronic supplementary material

ESM 1 (DOCX 47 kb)
