# Supplementary material for: Comparative clinical and cost analysis between surgical and non-surgical intervention for knee osteoarthritis
Source: Int Orthop. 2019 Sep 13;44(1):77–83. doi: 10.1007/s00264-019-04405-y (PMC6938792; doi:10.1007/s00264-019-04405-y)
Supplement: Supplementary file 1 — (DOCX 47 kb) [file 264_2019_4405_MOESM1_ESM.docx]

Appendix - costs

Table 1: Surgical Costs for Care of Osteoarthritis of the Knee

| **Procedure** | **Number of Procedures** | **Cost** | **Total Cost** |
| --- | --- | --- | --- |
| X-Ray | 6 (Pre-Op, Post Op, Day 3, Week 6, 1 year, Annually) | $31.95 | $192 |
| MRI | 2 | $62.80 | $125.60 |
| Blood Tests | 1 | $5.10 | $5.10 |
| Blood Test with Family Physician | 1 | $37.54 | $37.54 |
| ECG | 1 | $41.3 (Technical + Professional) | $41.30 |
| Scope | 1 | $97.35 | $97.35 |
|  |  |  |  |
| **Physician Consultations** | **Number of Visits** | **Cost** | **Total Cost** |
| Family Physician (Sports Medicine) | 6 (Pre and Post Operation, 2 weeks, 6 weeks, 3 months, 1 year, annually) | $33.70 | $202.20 |
| Orthopeadic Consultation | 1 | $83.10 | $83.10 |
| Surgical Consultation | 1 | $160.00 | $160.00 |
| Orthopaedic Repeat/Follow up Consultation | 4 (Post Op, 3 weeks, 6 months, 1 year) | $31.00 | $124.00 |
| Emergency Visit | 1 | $76.90 | $76.90 |
|  |  |  |  |
| **Operation Factors** | **Number of Uses** | **Cost** | **Total Cost** |
| Patellar Arthroplasty | 1 | $241.60 | $241.60 |
| Hemiarthroplasty - Total replacement both compartments | 1 | $619.90 | $619.90 |
| Hemiarthroplasty - Total replacement with take down of fusion | 1 | $838 | $838 |
| **Surgical Fee, Anasthesia Fee** | $7,675 | $7,675 | $7,675 |
|  |  |  |  |
| **Post-Operation Factors** | **Number of Times** | **Cost** | **Total Cost** |
| Cast | 1 | $24.10 | $24.10 |
| Application of Unna's Paste | 1 | $14.90 | $14.90 |
| Application of Cast Brace | 1 | $67.75 | $67.75 |
| Removal of Cast | 1 | $10.25 | $10.25 |
| Tylenol Arthritis | 24 tablets | $4.49 | $4.49 |
| Tylenol 3 | 24 tablets | $59.10 | $59.10 |
| NSAIDs (Motrin) | 6 weeks | $4.79 | $4.79 |
| Walker | 1 | $139.95 | $139.95 |
| Physiotherapy | Twice weekly, 6 weeks | $1,380 | $1,380 |
| Rehabilitation Hospital (semi-private) | 3 months | $150 | $13,500 |

Table 2: Non-Surgical Costs for Care of Osteoarthritis of the Knee

| **Procedure** | **Number of Procedures** | **Cost** | **Total Cost** |
| --- | --- | --- | --- |
| Family Physician | 1 | $76.90 | $76.90 |
| Family Physician | 1 | $33.70 | $33.70 |
| X-ray | 4 | $31.95 (technical + professional costs) | 127.8 |
| Family Physician Sports Medicine Referral | 2 (Once per year) | $33.70 | $67.40 |
| **Management – Procedure** | **Number of Procedures** | **Cost** | **Total Cost** |
| Injections (Depo Med + Lido 80g) |  | $22.05 | $22.05 |
| Apiration of Knee Joint |  | $39.80 | $39.80 |
| Depo-Medrol 80mg |  | $9.50 | $9.50 |
| Lidocaine (10x20mL) |  | $26.40 | $0.66 |
| Synvisc One |  | $143.43 | $143.43 |
| Durolane 20mg |  | $137.28 | $137.28 |
| Needle | pack of 50 | $15.00 | $0.33 |
| Gloves | pack of 100 | $12.00 | $12.00 |
| Alocohol Swabs | pack of 100 | $7.00 | $7.00 |
| Bandaids | pack 100 | $5.00 | $5.00 |
| Dictation | 2 months |  |  |
| Tylenol Arthritis | 24 tablets | $4.49 | $4.49 |
| Tylenol 3 | 24 tablets | $59.10 | $59.10 |
| NSAIDs (Motren) | 6 weeks | $4.79 | $4.79 |
| Knee Brace |  | $16.92 | $16.92 |
| Knee Support |  | $15.22 | $15.22 |
| Cane |  | $19.49 | $19.49 |
| **Physiotherapy** | Twice weekly, 6 weeks | $1,380 | $1,380 |
| Rehabilitiation Hospital semi-private | 3 months | $150 | $13,500 |
| Rehabilitiation Hospital private | 3 months | $250 | $22,500 |
| Rehabilitiation Hospital chronic | 3 months | $40 | $3,600 |
| Rehabilitiation Hospital chronic private | 3 months | $75 | $6,750 |
| **Rheumatology** | **Number of Times** | **Cost** | **Total Cost** |
| Management | 1 | $157.00 | $157.00 |
| Visit 1 | 1 | $79.85 | $79.85 |
| Visit 2 | 1 | $61.25 | $61.25 |
| Needle | 1 | $22.05 | $22.05 |
| Synovial Fluid Analysis | 1 | $24.70 | $24.70 |
